# Tools for assessing lung fluid in neonates with respiratory distress

**DOI:** 10.1186/s12887-022-03361-8

**Published:** 2022-06-20

**Authors:** So Jin Yoon, Jung Ho Han, Kee Hyun Cho, Joonsik Park, Soon Min Lee, Min Soo Park

**Affiliations:** 1 Department of Pediatrics, Yonsei University College of Medicine, Seoul, Republic of Korea; 2 Department of Pediatrics, Kangwon National University Hospital, Chuncheon, Republic of Korea

**Keywords:** Lung fluid, Neonate, Lung ultrasound, Electrical cardiometry, Cytokine

## Abstract

**Background:**

Transient tachypnea of the newborn (TTN), as a common cause of neonatal respiratory distress, needs to be distinguished from respiratory distress syndrome (RDS). Various modalities such as lung ultrasonography, cytokine analysis, and electrical cardiometry for the evaluation of lung fluid can be helpful for the exact diagnosis, however, clinical diagnosis has been applied mainly. This study aimed to evaluate the usefulness of the various tools for the diagnosis of TTN and RDS in neonates.

**Methods:**

This study evaluated 22 late-preterm and term infants admitted to the neonatal intensive care unit of Gangnam Severance Hospital because of respiratory distress. Total 9 neonates were diagnosed with TTN and 13 had RDS. In addition to chest radiography, the LUS score was calculated by a neonatologist using the portable ultrasound device. Cytokines in the bronchoalveolar lavage fluid supernatant were measured. Thoracic fluid content was measured using an electrical cardiometry device.

**Results:**

We enrolled 22 patients with median gestational age, 37.1 weeks, and birth weight 3100 g. There is no difference in patient characteristics between RDS and TTN group. Lung ultrasound score was significantly higher in RDS than TTN (11 vs 6, *p* = 0.001). Score 0 is shown in all infants with TTN. Score 1 is shown as significantly more in RDS than TTN. Between the TTN and RDS groups, there were significant differences in the changes of thoracic fluid content (2 vs − 1.5, *p* < 0.001), IL-1β levels (2.5 vs 11.3, *p* = 0.02), and TNF-α levels (20.1 vs 11.2, *p* = 0.04).

**Conclusion:**

We found lung ultrasound and electrical cardiometry to be reliable diagnostic tools for assessing infants with respiratory distress among late-preterm and term infants. Further studies with a large number of patients are needed to confirm their clinical usefulness.

## Background

Respiratory distress is one of the most common causes of admission to the neonatal intensive care unit (NICU), and failure to readily to recognize symptoms and to treat the underlying disease can lead to increased morbidity and mortality [1]. Transient tachypnea of the newborn (TTN) is the most common cause of neonatal respiratory distress in full term infants and is characterized by pulmonary edema resulting from delayed resorption and clearance of fetal alveolar fluid, and symptoms usually resolve spontaneously [2]. Respiratory distress syndrome (RDS) results from surfactant deficiency and an underdeveloped lung anatomy and requires early surfactant treatment. It occurs most often in babies born preterm while full term infants can also present with it but less frequently compared to preterm infants. The diagnosis of RDS is usually based on clinical manifestations and chest radiography findings [3]. In the clinical setting, it is important to differentiate between TTN and RDS.

Delayed resorption of fetal lung fluid because of differences between the oncotic pressure of air spaces, interstitium, and blood vessel is thought to be the underlying cause of TTN. The excess lung fluid in TTN results in decreased pulmonary compliance. Assessing the condition of lung fluid can be helpful for diagnosis and follow-up.

Lung ultrasound (LUS) has utility in the serial monitoring of the lung, especially with respect to pulmonary pathologies. The clinical application of LUS in newborns is expanding and promising. RDS has more severe clinical condition than TTN and has bilateral white lung finding in LUS diagnosis, so LUS is helpful tool for differentiating from other respiratory stress disease during neonatal period. Mixed TTN/RDS cases also exist and these cases are accompanied by partial surfactant deficiency that require positive pressure respiratory support or surfactant administration. Coppetti et al. [4, 5] reported that LUS showed high sensitivity and specificity in the diagnosis of transient tachypnea and RDS [6]. The LUS score has been reported showing significant correlations with oxygenation index [7]. LUS has also been reported to help reduce the use of surfactants for respiratory distress in late-preterm infants [8].

Some studies have demonstrated increased levels of pro-inflammatory cytokines in RDS. The pro-inflammatory cytokine, such as tumor necrosis factor (TNF), has been shown to contribute to the development of pulmonary edema and alveolar fluid reabsorption [9]. In addition, thoracic fluid content (TFC) using electric cardiometry has been postulated to provide an estimation of extravascular lung fluid [10], and TFC correlated well with extravascular lung fluid. TFC could be a useful tool for detecting pulmonary edema in mothers with pre-eclampsia and for diagnosing weaning failure in cardiac patients [10]. However, only a few studies have assessed lung fluid in neonates.

The purpose of this study was to assess lung fluid using LUS, cytokine analysis, and fluid measurement via electrical cardiometry for the differential diagnosis of RDS and TTN in late-preterm and full term infants.

## Methods

The study included 22 newborns between 34 and 40 weeks of gestation, who were admitted between January and December 2020 at the NICU of Yonsei University’s Gangnam Severance Hospital because of respiratory difficulty and evaluated lung fluid using LUS, cytokine analysis, and fluid measurement via electrical cardiometry. Gestational age was calculated based the best obstetric estimate using maternal last menstrual period or early pregnancy ultrasound examination findings or the new Ballard score if prenatal history is limited.

All newborns presented with respiratory difficulty including shallow breathing, chest retraction, tachypnea, and nasal flaring. RDS is diagnosed by three neonatologists based on the clinical manifestations, blood gas analysis, and chest X-ray findings after admission to the NICU. All patients diagnosed with RDS received surfactant through airway intubation. TTN is caused by delayed resorption of fetal lung fluid, and confirmed by chest x-ray with respiratory difficulty signs. Supportive care including supplemental oxygen, non-invasive and invasive ventilator care was provided for improving respiratory difficulty to the infants with TTN.

LUS was performed by a neonatologist using the portable ultrasound device, Vivid iq (General Electric Healthcare, Chicago, IL, USA). Using a transthoracic approach, with the patient in the supine position and with a 12-MHz linear probe, images were taken in three areas (anterior, lateral, and posterior) of both chests, and the collected images were analyzed. According to the study by Brat et al. [7], 0 points are assigned if line A is seen as a series of high-resolution horizontal equidistant lines below the pleura, 1 point if there were 3 or more B-lines per intercostal space, which are ‘comet tail’-shaped vertical lines starting from the pleural line, 2 points if ‘bilateral white lungs’ are observed, and 3 points if a large consolidation or pleural abnormality is observed. The LUS scores ranged from 0 to maximum 18 points.

Bronchoalveolar lavage (BAL) sample were collected when clinically indicated suctioning of the airway, using a standardized method. Aspiration of airway secretions was done using sterile traps with a suction catheter, and any material remaining in the catheter was washed into the trap with an additional 1 ml of saline. Samples were centrifuged at 14,000 g for 10 min, and the supernatant was frozen at − 80 °C (for 1–12 months) until assayed for cytokines [7, 11].

Cytokines in the BAL fluid supernatant were measured with ProcartaPlex 18-plex Immunoassay (eBioscience, San Diego, California, USA) on Qiagen Liquichip 200 (Qiagen, Hilden, Germany) running the Luminex 100 integrated system software (version 2.3; Luminex, Austin, Texas). The 18-plex cytokines included IL-1β, IL-2, IL-4, IL-5, IL-6, IL-7, IL-12, IL-13, IL-31, IL-1Rα, IFN-γ, GM-CSF, TNF-α, IFN-α, TNF-β, IL-1α, IL-15, and IL-18 The assay was done by the manufacturer’s protocol. Standard curves were constructed to interpolate analytes using ProcartaPlex Analyst version 1.0 (eBioscience). The mean of the technical duplicates was recorded.

Fluid Status using electrical cardiometry, ICON (Osypka Medical, San Diego, CA, USA) was shown as thoracic fluid content (TFC), stroke volume variation (SVV) and corrected flow time (FTC). TFC is derived from the thoracic electrical base impedance (1/base impedance), which is dependent on thoracic intravascular and extravascular fluid content. Larger TFC indicates a higher total thoracic fluid volume. TFC measurement has been correlated with heart failure symptoms, net fluid balance, and chest radiographic findings of abnormal pulmonary fluid content [12]. SVV is the percentage of change between the maximal and minimal stroke volumes. A greater SVV value represents intravenous volume depletion. FTC is the systole time divided by the square root of cardiac cycle time, which quantifies the time of blood flow through aortic valve and is an indicator of preload. After cleaning the neonate’s skin with alcohol, four electrocardiogram electrodes were placed at the neck below the left ear and just above the left clavicular midpoint, at the left midaxillary line – one at the level of the xiphoid process and the other electrode 5 cm below this point. The ICON device was connected to electrocardiogram, and measurement was continuously done for 30s, and the average of the highest and lowest values was recorded. Patient characteristics including gestational age, birth weight, sex, delivery method, multiple pregnancy, 1-minute/5-minute Apgar score, maternal conditions, and duration of respiratory support were collected from the electronic medical records.

The statistical analysis of the data was performed using IBM SPSS Statistics 20 (SPSS Inc., Chicago, IL, USA). Continuous variables are expressed as median (range) and were analyzed with the Mann Whitney U-test, and categorical variables are presented as numbers and percentages and were analyzed with the chi-square test. *P* < 0.05 was considered statistically significant.

The study was approved by the Ethics Committee of Gangnam Severance Hospital at the Yonsei University College of Medicine (IRB# 3–2019-0424). Research work was performed in accordance with the Declaration of Helsinki. A written informed consent was taken from the parents.

## Results

We enrolled 22 patients with median (range) gestational age, 37.1 weeks (34–40) and median birth weight, 3100 g (2290–3890). There was no difference of maternal history, respiratory parameters between RDS group and TTN group (Table 1).

**Table 1:** Patient characteristics of RDS and TTN groups

Variable	RDS (***n*** = 13)	TTN (***n*** = 9)	***P***-value
GA (weeks)	37.9 (34.4–39.7)	38.4 (34.3–40.0)	0.815
Birth weight (grams)	3030 (2290–3540)	3140 (2450–3890)	0.973
Multiple gestation	2 (16.5)	2 (22.2)	0.548
Oligohydramnios	1 (7.7)	1 (11.1)	0.789
PIH	1 (7.7)	2 (22.2)	0.340
PROM	6 (46.2)	3 (33.3)	0.557
Apgar score, 1 min	7 (6–8)	8 (7–9)	0.119
Apgar score, 5 min	8 (7–9)	9 (9–10)	0.142
Cesarean delivery	9 (69.2)	7 (77.8)	0.665
Male sex	6 (46.2)	5 (55.6)	0.672
Duration of ventilator care (days)	2 (1–3)	1 (0–2)	0.089
Duration of non-invasive ventilator care (days)	3 (1–5)	4 (2–4)	0.736
Duration of Oxygen care (days)	3 (2–4)	3 (2–4)	0.918
mortality	0 (0)	0 (0)	1.000

Values are expressed as median (range) or number (%). *Abbreviations*: *GA* Gestational age, *PIH* Pregnancy induced hypertension, *PROM* Premature rupture of membrane

Table 2 showed lung ultrasound findings in the infants between RDS and TTN. Lung ultrasound score was significantly higher in RDS than TTN. Score 0 (predominantly A-lines) was shown in all infants with TTN. Score 1 (mixed pattern with predominantly B-lines) was shown as significantly more in RDS than TTN, it means RDS group had more interstitial edema pattern in lung fields.

**Table 2:** Comparisons of LUS findings between RDS and TTN groups

Ultrasound findings	RDS (***n*** = 13)	TTN(***n*** = 9)	***P***-value
LUS total score	11 (10–12)	6 (5–8)	0.001
Predominantly A-lines	0 (0)	9 (100)	0.000
mixed pattern with predominantly B-lines	4 (30.8)	9 (100)	0.002
White lung appearance	10 (76.9)	0 (0)	0.001
Pleural line abnormalities	0 (0)	0 (0)	1.000
Large consolidation	1 (7.7)	0 (0)	0.405

Values are expressed as median (range) or number (%). *Abbreviations*: *TTN* Transient tachypnea of newborn, *RDS* Respiratory distress syndrome, *LUS* Lung ultrasound score

**Fig. 1:**
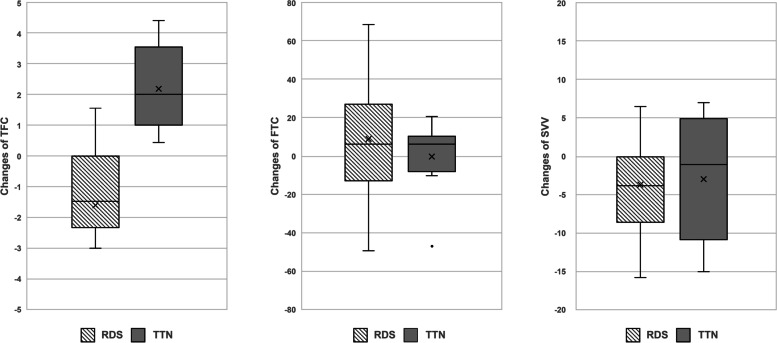
Comparisons of changes of TFC, changes of FTC, and changes of SVV measured from the mean first 6 hours of admission to the mean first 18–24 hours of admission

Table 3 showed fluid status-related parameters measured with the electrical cardiometry device between the RDS and TTN groups. Changes of TFC showed a significant increasing pattern in TTN during first 24 hours, whereas a decreasing pattern in RDS. However, changes of FTC and changes of SVV showed no significant differences (Fig. 1).

**Table 3:** Fluid status-related parameters measured with the electrical cardiometry device between the RDS and TTN groups

EC findings	RDS (***n*** = 13)	TTN(***n*** = 9)	***P***-value
TFC	25.75 (22.63–26.75)	23.50 (22.63–24.63)	0.404
Changes of TFC	−1.5 (−2.0 – −0.5)	2.0 (1.0–3.0)	0.000
FTC	278.13 (254.5–287.88)	288.75 (273.88–294.38)	0.182
Changes of FTC	6.5 (−6.5–+27.0)	6.0 (−3.5–+9.3)	0.593
SVV	18.63 (14.25–21.00)	17.63 (15.13–18.58)	0.689
Changes of SVV	−3.5 (−5.0 – − 0.5)	− 1 (−8.0–+4.5)	0.789

Values are expressed as median (range). *Abbreviations*: *TTN* Transient tachypnea of newborn, *RDS* Respiratory distress syndrome EC TFC, thoracic fluid content, *FTC* Corrected flow time, *SVV* Stroke volume variation

Table 4 showed the levels of cytokines in BAL samples. Only IL-1β level was significantly higher in infants with RDS than those with TTN (11.3 vs 2.5, *P* = 0.02). Only TNF-α level was significantly higher in infants with TTN than those with RDS (20.1 vs 11.2, *P* = 0.02). Other cytokines examined in this study showed no significant differences.

**Table 4:** Levels of cytokines in the bronchoalveolar lavage of neonates with RDS and TTN

	RDS (***n*** = 13)	TTN(***n*** = 9)	***P***-value
IL-1β (pg/mL)	11.3 (0.4–29.1)	2.5 (0.3–21.7)	0.02
IL-6 (pg/mL)	157.8 (51.4–615.4)	213.5 (43.2–443.8)	0.99
IL-8 (pg/mL)	870.8 (213.9–1517.9)	618.2 (125.9–1075.6)	0.07
IL-10 (pg/mL)	3.1 (0.6–5.2)	2.9 (1.4–4.6)	0.44
TNF-α (pg/mL)	11.2 (0.1–21.2)	20.1 (0.5–50.5)	0.04

## Discussion

The proper management of respiratory distress in neonates is important. This study demonstrated the usefulness of multiple methods for assessing lung edema for the differential diagnosis of RDS and TTN. Many studies have attempted to assess accuracy using only a single tool; however, multimodal assessment is the unique aspect of our study. Our technique can be a useful diagnostic tool for differentiating RDS from TTN.

TTN is a common cause of respiratory distress, with an incidence of 5.7 per 1000 births in the immediate newborn period. RDS is also observed in term or late preterm infants, but less frequently. Early diagnosis and accurate differentiation between RDS and TTN are of great importance, as RDS can be fatal without the appropriate treatment. According to the report from the United States, the incidence of RDS in preterm infants in 2010 was 1.6–5.4% [13]. According to a multicenter study in Korea in 2007, RDS was noted in 10–20% of infants with a gestational age of 33–34 weeks, and in less than 5% in infants with a gestational age of 35–36 weeks [14]. In a nationwide epidemiologic study in Korea, RDS was noted in 0.25% of infants with a birth weight of ≥2500 g and in 5.1% of infants with a birth weight of 1500–2500 g, with an increase of 9.5% from 2014 to 2018 [3]. Thus, it seems necessary to determine the best way for differentiating between RDS and TTN.

LUS has been used to diagnose lung diseases in newborns with high accuracy, reliability, and simplicity and with no risk of radiation injury [15]. In the differential diagnosis of RDS and TTN, LUS showed high sensitivity and specificity [6]. Neonates with RDS had higher LUS scores than those without, and the LUS scores increased with RDS severity [16, 17]. The LUS score for the prediction of RDS showed 80.2% sensitivity and 100% specificity using a cut-off of 21.5 (AUC = 0.938) [16]. LUS showed better performance in the diagnosis of RDS; for diagnosing RDS with pooled sensitivity of 0.99 and a specificity of 0.95 and for TTN with pooled sensitivity of 0.67 and specificity of 0.97 [18].

In addition, LUS showed a good correlation with oxygenation indices and good reliability for predicting surfactant administration in preterm neonates under continuous positive airway pressure [7]. Moreover, in extremely preterm neonates with RDS, LUS scores can be used to predict the need for the first surfactant dose and the need for surfactant redosing [19]. A single-center study in Korea showed that LUS helped to reduce the use of surfactants from 12.3 to 9.4% in preterm infants at 34–36 weeks gestation [8]. In this study, we confirmed the findings again that the LUS score is significantly different between RDS and TTN.

TTN is a parenchymal lung disease characterized by pulmonary edema caused by delayed absorption and removal of fetal alveolar fluid [20]. The accumulation of fluid in the peribronchiolar lymphatics and interstitium promotes the partial collapse of the bronchioles with subsequent air trapping [21]. Highly permeable pulmonary edema usually refers to direct or indirect damage to the capillary endothelium in the lungs. Indirect injuries commonly associated with blood-borne mediators, such as leukotrienes, histamine, bradykinin, or some pro-inflammatory cytokines, including IL-1β, IL-6, IL-8, IL-10, and TNF-α [22–24]. Some studies suggest that cytokines play an important role in the development of pulmonary edema. In a male neonate with pulmonary edema, the cytokines IL-6, IL-8, IL-10, IL-13, IL-17, and IFN-γ rises sharply during the first few hours after birth and then decreases significantly [25]. TNF, the most widely studied pleiotropic cytokine [11], seems to have a dual role, which means it does not only promote the regression of pulmonary edema, it also helps to stimulate lung fluid clearance [14]. In this study, we could see a higher level of TNF-α in BAL fluid of TTN babies than RDS infants (*p* = 0.04). On the other hand, RDS is caused by a lack of surfactant proteins in the lungs in addition to early inflammatory responses [26]. Cytokines and other inflammatory mediators can cause the dose-dependent suppression of surfactant proteins in the perinatal period [5]. Previous study showed that levels of the proinflammatory cytokines IL-8 and IL-1β were significantly higher in BAL of infants with severe RDS than those with moderate RDS [26]. Very low birth weight infants have increased concentrations of the proinflammatory cytokines IL-8, IL-1β, IL-6 and MCP-1 in tracheal aspirates and their levels were associated with the risk of bronchopulmonary dysplasia [27]. The inhibitory cytokine, IL-10, downregulates inflammation in part by reducing the production of the proinflammatory cytokines and can be detectable in early BAL samples from preterm infants [28]. In our study, infants with RDS had definitely higher values of IL-1β and IL-8 than those with TTN, though IL-8 showed only a high correlation (*p* = 0.07). This can be one of the evidences of the early inflammation in RDS and shows the same context as previous studies. Since both RDS and TTN are associated with proinflmmatory cytokines, some of them may not differ between the two groups, like IL-6 and IL-10 in our study. However, since there is a clear difference in the developing mechanisms of the two diseases, finding and measuring the cytokines in tracheal aspirates that show differences between two groups would be one of the simple and easy ways for diagnosis and prognostication. Further analysis with more samples would be needed.

Electrical cardiometry has been proposed as a safe, accurate, and reproducible technique for hemodynamic measurement in children and infants. TFC correlated well with ultrasound in the estimation of extravascular lung fluid [29], ranging from 15.1 in preterm infants to 41.4 in term infants. The high TFC value could in-directly reflect lung congestion or hypervolemia, as a known risk factor for weaning failure [10]. TFC correlated both with the presence of respiratory distress and with its resolution in newborns with TTN [30]. TFC has been used to assess the hemodynamic effect of diuretics and to evaluate thoracic fluid in patients with heart failure [31]. TFC has also shown good correlation with fluid balance during cardiac surgery [32]. In this study, TFC was significantly higher in the TTN group than in the RDS group, in accordance with the newborn reference range. By confirming that TFC increased over time in TTN, it was possible to confirm the developing mechanism of TTN caused by excess lung fluid and pulmonary edema. In other words, even TTN and RDS infants shows similar respiratory difficulties or chest x-ray findings, the electrocardiometry can help us to distinguish between the two groups in the actual clinical fields.

This study was limited by its small sample size because of the limitation of evaluating lung fluid using LUS, cytokine analysis with BAL aspirates, and fluid measurement via electrical cardiometry simultaneously. Since not all the TTN and RDS infants corresponding to the inclusion criteria could be assessed, there might be a selection bias, which was also a major limitation and would be solved by collecting more data and continuing the subsequent study. The time points of evaluation can be variable within first 24 hours of age, so it can be influenced to the results. No adjusted or blinded assessments related to bias were performed.

## Conclusions

We found that LUS, electrical cardiometry and cytokine analysis can be used for differentiating RDS and TTN in the clinical fields. In particular, LUS and electrical cardiometry represent attractive and easy-to-use bedside diagnostic tools for assessing infants with respiratory distress. Further studies with a large number of patients are needed to confirm their clinical usefulness.

## Data Availability

The datasets generated and/or analyzed during the current study are not publicly available due to ethical restrictions but are available from the corresponding author on reasonable request.

## Abbreviations

FTC: Corrected flow time
LUS: Lung ultrasound
RDS: Respiratory distress syndrome
SVV: Stroke volume variation
TFC: Thoracic fluid content
TNF: Tumor necrosis factor
TTN: Transient tachypnea of the newborn
BAL: Bronchoalveolar lavage
